# Sub-cellular proteomics of *Medicago truncatula*

**DOI:** 10.3389/fpls.2013.00112

**Published:** 2013-04-30

**Authors:** Jeonghoon Lee, Zhentian Lei, Bonnie S. Watson, Lloyd W. Sumner

**Affiliations:** Plant Biology Division, The Samuel Roberts Noble FoundationArdmore, OK, USA

**Keywords:** *Medicago truncatula*, subcellular proteomics, mass spectrometry based proteomics, legumes, nodulation, arbuscular mycorrhizal symbiosis

## Abstract

*Medicago truncatula* is a leading model species and substantial molecular, genetic, genomics, proteomics, and metabolomics resources have been developed for this species to facilitate the study of legume biology. Currently, over 60 proteomics studies of *M. truncatula* have been published. Many of these have focused upon the unique symbiosis formed between legumes and nitrogen fixing rhizobia bacteria, while others have focused on seed development and the specialized proteomes of distinct tissues/organs. These include the characterization of sub-cellular organelle proteomes such as nuclei and mitochondria, as well as proteins distributed in plasma or microsomal membranes from various tissues. The isolation of sub-cellular proteins typically requires a series of steps that are labor-intensive. Thus, efficient protocols for sub-cellular fractionation, purification, and enrichment are necessary for each cellular compartment. In addition, protein extraction, solubilization, separation, and digestion prior to mass spectral identification are important to enhance the detection of low abundance proteins and to increase the overall detectable proportion of the sub-cellular proteome. This review summarizes the sub-cellular proteomics studies in *M. truncatula*.

## INTRODUCTION

Proteomics has become an important research tool to study complex biological systems in the post-genomics era, and the large-scale, systematic analysis of tissue and organelle specific proteins provides a more direct view of cellular processes not available through the measurement of DNA. Proteomics can provide insight on the specialized biochemistry of distinct tissues, protein localization, protein–protein interactions, enzymatic complexes, protein-metabolite complexes, post-translational modifications, and cellular signaling (Kersten et al., 2002; Baginsky, 2009). There are two general strategies for large-scale proteome analysis: bottom-up and top-down. With the bottom-up approach, complex protein mixtures are digested and resultant peptides analyzed by mass spectrometry (MS) for protein identification and quantification. Often the complex mixtures are purified using electrophoretic or chromatographic separations to render enriched or purified proteins which are then subjected to proteolytic digestion and mass spectral protein identification. One example would be two-dimensional polyacrylamide gel electrophoresis used for separation of complex protein mixtures followed by proteolytic digestion of isolated spots and mass spectral fingerprinting (Egelhofer et al., 2002). An alternate, “shotgun” proteomics approach to bottom-up sequencing uses enzymatic digestion of complex mixtures and multi-dimensional separations, such as ion exchange chromatography and high performance liquid chromatography, of the proteolytic fragments (Motoyama et al., 2006). Mass spectrometric peptide mapping and database searching is then performed.

Progress in the area of proteomics has relied heavily on the development of analytical tools for the sensitive, selective, and high-throughput studies of protein analytes (Aebersold and Goodlett, 2001). MS has evolved into a primary analytical tool for proteomics research, especially when coupled with high resolution separation techniques, due to the high information content that can be derived from these coupled techniques (Aebersold and Mann, 2003). Advances in MS have been substantially facilitated by two ionizations techniques; electrospray ionization (ESI) and matrix-assisted laser desorption/ionization (MALDI). Over the course of the past two decades, these ionization methods have become indispensable for the analysis of biological molecules, especially proteins and peptides. ESI MS produces highly charged ions directly from liquids and is therefore useful for coupling to liquid separations (Fenn et al., 1989; Griffiths et al., 2001; Domon and Aebersold, 2006). MALDI is fast and efficient and has a high tolerance to non-volatile buffers and impurities (Hillenkamp et al., 1991; Hardouin, 2007). The samples for MALDI are typically applied to solid supports and used off-line from liquid or gel separations (Walker et al., 1995; Rejtar et al., 2002).

Separation of the proteome is challenging due to the complexity of the proteome. Through post-translational modifications and differential splicing, 5–10 different protein variants can often be produced from each gene (Collins et al., 2004; Fröhlich and Arnold, 2006). A further complication is the dynamic range of protein expression at the cellular level, which can range from one up to 10^6^–10^9^ copies per cell (Corthals et al., 2000). Many important proteins are present at low abundance and are difficult to isolate from complex mixtures containing more highly abundant proteins with current separation methods (Hancock et al., 2002; Hunter et al., 2002). As an example, two-dimensional gel electrophoresis (2-DE) can separate up to 11,000 proteins from a whole cell lysate, but is restricted to the most highly abundant proteins in the sample (Gygi et al., 2000). High peak capacity separations of proteins with better resolution and faster analysis times are required for continued improvement of proteomic analyses. This can be accomplished in a bottom-up fashion, in which the proteins are proteolytically digested into peptide fragments and separated before MS analysis.

**FIGURE 1:**
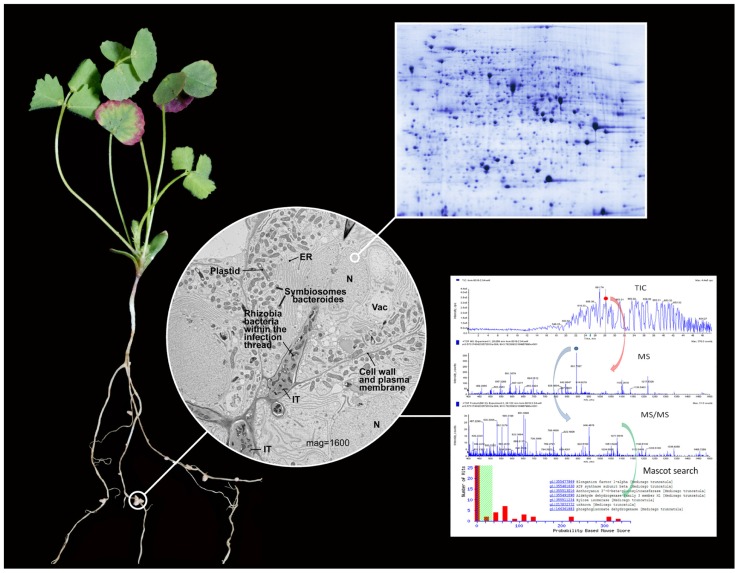
**Illustration of the sub-cellular proteomics work flow**. A 4-week old *Medicago truncatula* R108 plant is shown with root nodules 18 days post inoculation with *S. meliloti*. The root nodules are specialized root organs resulting from the symbiotic infection of legumes with soil rhizobia bacteria that enable nitrogen fixation. Symbiotic nitrogen fixation provides a ready supply of nitrogen to the plant and carbon to the bacteria, and results in plants with high protein content. Thus, legumes serve as important nutritional resources throughout the world for humans and animals. The central micrograph shows the cellular components of *M. truncatula* root nodule during an early stage of infection and at a magnification of 1600. Clearly visible and labeled are traditional organelles such as the nucleus (N), vacuole (Vac), endoplasmic reticulum (ER), plastid (P) containing starch grains, and cell wall/plasma membrane. The micrograph provides an image of the rhizobia infection thread (IT) which results from the invagination of the cell wall/plasma membrane and contain rhizobium bacteria. Bacteroides are formed as the bacteria segregate from the infection thread and enter the cell and are encapsulation by a symbiosome plant membrane. The number of bacteroides generally increases with the maturity of the nodule cell. Many of the sub-cellular organelles and especially those related to symbiosis have been isolated and analyzed using various proteomics methods such as 2-DE or LC-MS/MS to better understand the protein composition and function of these specialized organelles. An example of a nano LC-MS/MS shotgun proteomics experiment is provided that includes the total ion chromatogram (TIC), full scan mass spectrum (MS), and tandem mass spectrum (MS/MS). These data are used to search predicted proteins from DNA or RNA or sequenced protein databases to identify proteins observed in the LC-MS/MS experiment. The *M. truncatula* micrograph was provided by Dr. Jin Nakashima, Manager of the Noble Foundation Cellular Imaging Facility, and Jiejan Xi. The plant photo was provided by Mr. Broderick Sterns. The representative 2-DE and LC-MS/MS images were provided by the authors.

Legumes are economically valuable crops in the United States, Asia, South America, and throughout the world. Legumes form unique symbiotic relationships with nitrogen-fixing soil bacteria such as *Rhizobia* which provide nitrogen to the plant (Sprent, 2007). The accumulation of high protein levels in legumes provides an economical dietary source of proteins for humans and animals. *Medicago truncatula* (**Figure 1**) has been utilized as a model legume because of its small diploid genome compared to other legumes which have large, complex polyploidy genomes (Bell et al., 2001). It also has a short generation time, prolific seed production, and good transformation efficiency (Cook, 1999; Trieu et al., 2000). Many molecular, genetic, and biochemical resources are now available for *M. truncatula,* and it is a mature model for the study of legumes and legume biology. Legumes are also a rich source of a wide variety of natural products such as flavonoids, isoflavonoids, saponins, and alkaloids which have potential in pharmaceutical and biotechnological applications (Dixon and Sumner, 2003). Many natural products are produced in specific tissues such as glandular trichomes and/or stored in sub-cellular vacuoles.

Cellular proteins are actively and passively transported across cellular and organelle membranes during normal homeostasis and in response to stress and external stimuli (Agrawal et al., 2005a,b). Accurate identification and quantification of a sub-cellular proteome is very useful and can provide insight into cellular/organelle function and dynamics (Lilley and Dupree, 2007). In addition to the field of functional proteomics, sub-cellular proteomics can provide insight into the molecular mechanisms of plant cell modulation of protein accumulation in intracellular compartments in response to various perturbations, and thus provides refined knowledge about signal transduction in organelles (Hossain et al., 2012). In order to achieve an accurate description concerning the sub-cellular localization of specific proteins, rigorous isolation procedures are important to prevent cross contamination from other cellular and organelle proteins. Some of the major sub-cellular organelles are nucleus, mitochondria, chloroplasts, plastids, peroxisomes, plasma membrane, cytosolic ribosome, and extra cellular structures. The proteomes of multiple tissues, sub-cellular organelles, and membrane systems have been describe from * M. truncatula* (**Figure 1**). Several of the global proteomics approaches for protein characterization from different tissues/organs of *M. truncatula* were based on 2-DE and MS (Mathesius et al., 2001; Watson et al., 2003). These studies focused upon total protein characterization but the analytical tools are still useful for the analysis of proteins from individual cellular compartments (Valot et al., 2006; Soares et al., 2007). The combination of protein separation by one-dimensional electrophoresis with LC-MS/MS has also been utilized for identification of differentially expressed sub-cellular proteins (Lefebvre et al., 2007; Daher et al., 2010). Due to hydrophobic nature of most membrane proteins, gel-free LC-MS/MS or multi-dimensional protein identification technology (MudPIT) systems have also been used to investigate sub-cellular membrane components (Zhang et al., 2006).

In this article, the various proteomic studies focused on sub-cellular organelles of *M. trucatula* are briefly reviewed. Several analytical technologies employed to study specific *M. trucatula* sub-cellular compartments are also presented.

## *M. truncatula* SUB-CELLULAR PROTEOME

The plant nucleus is of great interest because it contains the genomic content and is the critical site of transcription and replication in eukaryote cells. Repetto et al. (2008) used proteome analyses to identify nuclear regulators of developing *M. truncatula* seeds and to understand the molecular mechanisms of seed development. The purity of the nuclear preparations were assessed by Western blot analyses using antibodies directed against histone H1, uridine diphosphate (UDP)-glucose pyrophosphorylase, vacuolar ATPase, photosystem II reaction center protein D1, and mitochondrial porin. A total of 179 peptides from 143 different proteins were identified using nano LC-MS/MS analysis of one-dimensional SDS-PAGE in-gel digests obtained from the seed nuclear proteome at 12 days after pollination (dap) which marks the switch to seed filling. Identified proteins were associated with roles in biogenesis of ribosomal subunits or nucleocytoplasmic trafficking. The results showed that 12-dap seeds accumulated ribosomal proteins in preparation for protein synthesis activity prior to seed filling. Other identified proteins were related to chromatin structure, transcription, RNA maturation, silencing, and transport to regulate gene expression and seed development.

Mitochondria are semiautonomous organelles involved in energy metabolism. They are also involved in many other cellular functions including amino acid and nucleotide metabolism, synthesis of cofactors, and photosynthesis. The majority of mitochondrial proteins involved in different metabolic processes are encoded by nuclear DNA, translated in the cytosol, and imported into mitochondria. Dubinin et al. (2011) isolated *M. truncatula* mitochondrial fractions from root cell suspension cultures using Percoll gradient ultracentrifugation. The mitochondrial proteome of *M. truncatula* was characterized using 2-DE IEF/Tricine SDS-PAGE and 2-DE blue-native (BN)/Tricine SDS-PAGE. A total of 144 proteins were identified by 2-DE IEF/SDS-PAGE and 51 proteins were identified on the 2-DE BN/SDS-PAGE. The identified proteins were related to oxidative phosphorylation (OXPHOS), pyruvate decarboxylation and citric acid cycle, amino acid degradation, and ATP synthesis. The *M. truncatula* 2-DE mitochondrial proteome maps revealed similarities to the one from *Arabidopsis*. However, the abundance of complex II was increased in *M. truncatula* compared to *Arabidopsis*, which indicates increased citric acid cycle activity in *M. truncatula *mitochondria. Highly abundant prohibitin complexes were also present in the mitochondrial proteome of *M. truncatula*.

Intercellular fluid (IF) and ionically bound (IB) proteins of *M. truncatula* leaf apoplast were analyzed using 2-DE separation coupled with MALDI-TOF/TOF MS identifications (Soares et al., 2007). The apoplast contains proteins of various functions including cell expansion, growth cessation, signaling, and response to biotic and abiotic stresses. Compared to other sub-cellular proteomics reports, a lower number of proteins from apoplast were characterized because of the difficulty in isolating apoplastic proteins free from intracellular contamination. Proteins were extracted with a sodium acetate buffer and were isolated by centrifugation followed by pressure-assisted filtration using molecular weight cut-off membrane filters. Soares et al. (2007) separated 220 IF and 84 IB proteins in the *M. truncatula* leaf apoplast. Malate dehydrogenase activity was measured as a marker of cytosolic contamination from leaf extracts. An analysis of 2-DE of apoplastic proteins revealed the IF and IB proteins consisted largely of different protein populations representing distinct functional components of the apoplast. The authors found a high number of chitinases and β-1,3-glucanases which are glycine-rich proteins associated with defense and predominate the IF. Identified IB proteins were related to energy production/conversion, oxidoreductases, transport/binding of solutes, and cell wall structure.

Extracellular and secreted proteins (cumulatively referred to as the secretome) serve important roles in intercellular communication, development, and defense. Many secreted proteins are found in the apoplast. However, isolating proteins from apoplast is challenging and often contaminated with intracellular proteins as noted above. To circumvent intracellular contamination and to better understand apoplast protein composition, Kusumawati et al. (2008) proposed cell cultures as a model system to study apoplast proteins and characterized the secreted proteins from the extracellular medium of three *Medicago* cell suspension cultures. These included *M. truncatula *2HA, *M. truncatula sickle*, and *M. sativa* cell lines. The authors suggest that cell suspension cultures provide an effective method to obtain representative apoplastic proteins without damaging the plant and potentially little or no sample contamination with cytoplasmic proteins. The *M. truncatula* cell culture secretome was isolated using a two-step centrifugation process. A total of 26 proteins were identified in the cultures derived from *M. truncatula* using SDS-PAGE and MALDI-TOF/TOF MS. Among the identified proteins, three secreted proteases including a subtilisin-like serine protease, aspartyl protease, and a serine carboxypeptidase were identified and detected only in *M. truncatula* cell cultures and not in the other Medicago cell cultures. Twelve putative defense response proteins including four chitinases, a peroxidase, three thaumatin-like PR5 proteins, PR1a, PR4a, and a prehevein-like protein were also identified from *M. truncatula* secretome. The authors conclude that all the identified cell culture secreted proteins are part of the classical or non-classical secretion pathways and have not been reported in other *M. truncatula* tissues. Therefore, they further conclude that cell culture secreted proteins are a good approximation of the appoplastic proteins of *Medicago* spp.

Plant membranes are key in maintaining homeostasis and in the transport/storage of cellular materials. They are also important in the perception and transduction of signals that enable effective communication with the cell’s surroundings. Valot et al. (2004) reported on the total root microsomal proteome from *M. truncatula* due to the importance of this model legume in studying plant–microbe interactions; especially symbiosis. Microsomal proteins were separated using 2-DE, and three different extraction methods were compared for preparing microsomal proteins. The extraction methods were based on phenol, acetone, and a chloroform/methanol mixture. A total of 440 microsomal proteins were visualized using analytical, silver stained 2-DE. Ninety-six proteins from 115 Coomassie stained micropreparative 2-DE protein spots were identified following in-gel digestion, MALDI-TOF MS peptide mass fingerprinting, and *M. truncatula* clustered EST database searches. This search method led to an increase in the percentage of successfully identified proteins to 83%. The Valot et al. (2004) report provided early methods for *M*. *truncatula* membrane proteomics and a catalog of membrane proteins useful to future studies of these important cellular components.

Lefebvre et al. (2007) investigated the role of lipid rafts from plasma membranes of *M. truncatula* and provided an extensive analysis of their structure, lipid composition, and protein content. In the process, the authors identified 270 proteins associated with *M. truncatula* lipid rafts. The contamination of the plasma membranes was evaluated through the measurement of azide-sensitive ATPase activity for mitochondria and nitrate-sensitive ATPase for tonoplasts. The major proteins found were related to signaling, transport proteins, redox proteins, cytoskeleton, trafficking, and protein stability. The authors conclude that the lipid rafts contain a complete plasma membrane redox system important for stress mediation and defense, and contain a large number of receptor-like protein kinases (RLKs) that re-enforce the hypothesis that plant lipid rafts are part of the signaling network similar to animals.

The sub-cellular proteomes of membrane-associated protein modifications in response to arbuscular mycorrhizal (AM) symbiosis have also been studied (Valot et al., 2005). This early study focused on the identification of AM fungal proteins in planta. Total membrane fractions were obtained by differential centrifugation. Membrane proteins were then extracted with a cold mixture of chloroform/methanol (6/3, v/v). Membrane proteins were extracted from AM fungal (*Glomus intraradices*) inoculated or non-inoculated *M. truncatula* roots. Thirty-six 2-DE protein spots were differently accumulated where 15 proteins were induced, three were up-regulated, and 18 were down-regulated compared to 2-DE protein spots from non-inoculated roots. Among them, 25 spots were identified with MALDI-TOF MS peptide mass fingerprinting. Most of the identified proteins had not been associated with AM symbiosis previously via proteomics, transcriptomics, nor or suppressive subtractive hybridization which illustrated the potential of direct proteomics. Nineteen of the 25 mycorrhiza-responsive proteins were found to be not regulated by phosphate supply further suggesting that these proteins could serve as markers for AM symbiosis.

Valot et al. (2006) followed with another report focused upon the study of periarbuscular membrane composition which identified 78 proteins from enriched membrane fractions obtained using a discontinuous sucrose gradient method. Marker enzymes for the plant cell membranes were assayed using K^+^, Mg^2^^+^-ATPase sensitivity assays. Pyrophosphatase, inosine diphosphatase, nicotinamide adenine dinucleotide (NADH)-cytochrome c reductase insensitive to antimycin A and cytochrome c oxidase were used for markers for tonoplast, golgi, endoplasmic reticulum, and mitochondria respectively. In this study, the authors used two-dimensional LC-MS/MS or gelC-LC-MS/MS for protein separation and identification as opposed to the 2-DE method used in their earlier report which allowed for the identification of a larger number of proteins (Valot et al., 2005). Comparison between *G. intraradices* inoculated and uninoculated membrane fractions revealed two differentially accumulated proteins; i.e., H^+^-ATPase (Mtha1) and a predicted glycosylphosphatidylinositol-anchored blue copper-binding protein (MtBcp1). The role of these proteins in AM symbiosis remains to be investigated.

Symbiosome membrane proteins from *M. truncatula* and the corresponding *Rhizobia* bacterium *Sinorhizobium meliloti* were investigated using 2-DE and LC-MS/MS (Catalano et al., 2004). Symbiosome membranes derived from the plant plasma membrane encircle *Rhizobia* in infected root nodule cells. See **Figure 1**. This membrane is important for biological nitrogen fixation. Proteins in the symbiosome membrane are critical for transport, energy, metabolic processes, nodule formation, signaling, pathogen response, and protein destination. Symbiosomes were isolated from *M. truncatula* root nodules using differential centrifugation. Western blot analyses were used to evaluate the fraction purity by comparison of protein distributions between 2-DE of symbiosome membrane and other symbiosome fractions. Fifty-one proteins, mostly associated with protein destination and storage, were identified from the symbiosome membrane. Twenty-eight plant symbiosome proteins were functionally classified into energy and transport, protein destination, nodule-specific, and unclassified categories. The proteomics results provide a better defined biochemical composition of the symbiosome membrane and serve as a hypothesis generating dataset to better understand the mechanism of plant–rhizobia symbiosis.

Larrainzar et al. (2007) characterized the proteome of *M. truncatula* root nodules in response to drought stress. Proteins from nodules of *M. truncatula* in symbiosis with *S. meliloti* were profiled using fast protein liquid chromatography (FPLC) and two-dimensional LC-MS/MS. Western blot analyses were performed using antibodies against NifDK as a marker for bacteroid contamination. A quantitative analysis of plant and bacteroid responses to drought stress was also performed using one-dimensional-LC-MS/MS. The larger identified functional protein classes were involved in protein synthesis and degradation, amino acid metabolism, and glycolytic pathway and TCA cycle. The other functional groups comprised proteins involved in redox state control, defense against biotic and abiotic stress, and signaling processes in nitrogen-fixing nodules. In the quantitative study, the authors found the relative content of five nodule proteins such as Met synthase, SuSy, Asn synthetase (AS), Lb, and the transcriptional eukaryotic elongation factor-2 (eEF-2) decreased following water deficit compared to those from control nodules. The data confirm the role of SuSy and identified four new enzymes involved in drought stress.

Daher et al. (2010) studied the root plastid proteome using nano LC-MS/MS and identified 266 protein candidates which have a role in nucleic acid-related processes, carbohydrate, and nitrogen/sulfur metabolisms, and stress response mechanisms. A major challenge to root plastid proteomics was the isolation of the colorless, low abundant, heterogeneous, and fragile organelles which was ultimately achieved using a differential centrifugation method originally developed for pea (*Pisum sativum*). Another challenge was the identification of a whole set of proteins with diverse chemical properties. The structures of non-photosynthetic plastids are important because they are associated with nitrogen-assimilation, starch, and lipid synthesis. Plastids are also involved in connecting individual organelles during symbiotic nitrogen fixation. In this article, the authors provide an impressive comparison of the *M. truncatula* root plastid proteome relative to the proteomes of amyloplasts, chloroplasts, and proplastids. The authors conclude that the functional distribution of the *M. truncatula* root plastids proteome mainly resemble that of wheat endosperm amyloplasts and tobacco proplastids, but are markedly different from chloroplasts.

## CONCLUSION

Sub-cellular proteomics, the analysis of proteins purified from a cell compartment, has emerged as a promising method to better understand the spatial segregation of cellular processes and organelle function. A growing number of studies of plant proteomics have been reported on elucidating the protein functions and dynamics of plant sub-cellular compartments. Although more extensive sub-cellular proteomics literature exists for *Arabidopsis* and rice, research using *M. truncatula* as a model system is increasing with the bulk of these efforts focused upon plant–microbe interactions and symbiosis. The current *M. truncatula* sub-cellular proteomics literature reveals nuclear proteome contains specific proteins involved in signaling and gene regulation; whereas the mitochondrial and chloroplast proteomes revealed protein fractions implicated in energy production, either in electron transport or in ATP production. Additional studies on the apoplast or secreted proteomes revealed predominantly defense related and cell wall modifying proteins. Substantial effort has also been focused on *M. truncatula* membranes during mutualism with AM symbiosis and many of the identified proteins had not been associated with AM symbiosis previously thereby providing additional compositional information relative to this important plant–fungal interaction. Legumes are unique in their ability to form unique symbiotic relationships with soil rhizobia in the fixation of nitrogen. Identified proteins in the* M. truncatula* rhizobia symbiosome membranes are critical for transport, energy, metabolic processes, nodule formation, signaling, pathogen response, and protein destination. These results provide novel insight into the exchange of nutrients and plant–rhizobia communication.

Overall the number of *M. truncatula *sub-cellular identified proteins is still quite modest relative to the number of proteins expected to be present and relative to other plant species such as *Arabidopsis* and rice. More comprehensive sub-cellular proteomics analyses; especially related to symbiosis, are expected to be even more informative. The accurate characterization of the proteome in different sub-cellular locations also remains a challenging task dependent on the successful isolation of purified organelles. The extent of error associated with the interpretation of protein sub-cellular localization is often due to inevitable contamination from other sub-cellular components. Accurate methods to detect and quantify proteins in mixtures are necessary to assess the purity of sub-cellular purification. Although the analysis of marker enzymes based on organelle specific antibodies and the microscopic detection of sub-organelle fractions are still useful to determine the purity, the accuracy is low and varies considerably. However, the combination of 2-DE profiling, MS-based measurements, and the use of genomic data can be useful for comparing protein identities and abundances among samples. In addition to developments in sub-cellular isolation methods, advances in bioinformatics are still needed to enable large-scale comparison and correlation of large datasets from various sub-cellular proteomics studies to better describe complex biological system.

## Conflict of Interest Statement

The authors declare that the research was conducted in the absence of any commercial or financial relationships that could be construed as a potential conflict of interest.
